# Supplementary material for: Comparative Expression Profiling Reveals the Regulatory Effects of Dietary Mannan Oligosaccharides on the Intestinal Immune Response of Juvenile Megalobrama amblycephala against Aeromonas hydrophila Infection
Source: Int J Mol Sci. 2023 Jan 22;24(3):2207. doi: 10.3390/ijms24032207 (PMC9917204; doi:10.3390/ijms24032207)
Supplement: Supplementary file 1 [file ijms-24-02207-s001.zip › supplementary Table S1.pdf]

International Journal of
Molecular Sciences

Supplementary Table S1. Ingredients and nutrient composition of the experimental diets.

Ingredients	Control (%)	MOS200 (%)	MOS400 (%)
Fish meal	8.00	8.00	8.00
Soybean meal	20.80	20.80	8.00
Cottonseed meal	15.00	15.00	15.00
Rapeseed meal	18.00	18.00	15.00
Wheat middling	30.00	30.00	30.00
Soybean oil	5.00	5.00	5.00
$Ca(H_2PO_4)_2$	2.00	1.98	1.96
MOS	-	0.02	0.04
Choline	0.30	0.30	0.30
Vitamin premix[1)]	0.40	0.40	0.40
Mineral premix[2)]	0.50	0.50	0.50
Total	100	100	100
Nutrient levels[3)]			
Moisture	6.88	6.89	6.91
Crude protein	37.10	36.78	36.91
Crude lipid	8.43	8.41	8.28
Ash	7.50	7.51	7.51

1) Vitamin premix for each kg of feed: VE 50 mg, VA 5 000 IU, VB1 8 mg, VK 5 mg, VB6 8 mg, VD 2 000 IU, VB2 10 mg, Pantothenic acid 30 mg, VB12 0.03 mg, Folic acid 3 mg, Niacin 30 mg. mg, inositol 100 mg, biotin 0.4 mg, VC 180 mg.
2) Mineral premix for each kg of feed: Mg 300 mg, Zn 150 mg, Fe 170 mg, Co 0.25 mg, Cu 4 mg, Mn 22 mg, Se 0.4 mg.
3) Moisture, crude protein, crude lipid and ash contents were Calculated values.
