# Supplementary figures and images for: Comparative Expression Profiling Reveals the Regulatory Effects of Dietary Mannan Oligosaccharides on the Intestinal Immune Response of Juvenile Megalobrama amblycephala against Aeromonas hydrophila Infection
Source: Int J Mol Sci. 2023 Jan 22;24(3):2207. doi: 10.3390/ijms24032207 (PMC9917204; doi:10.3390/ijms24032207)

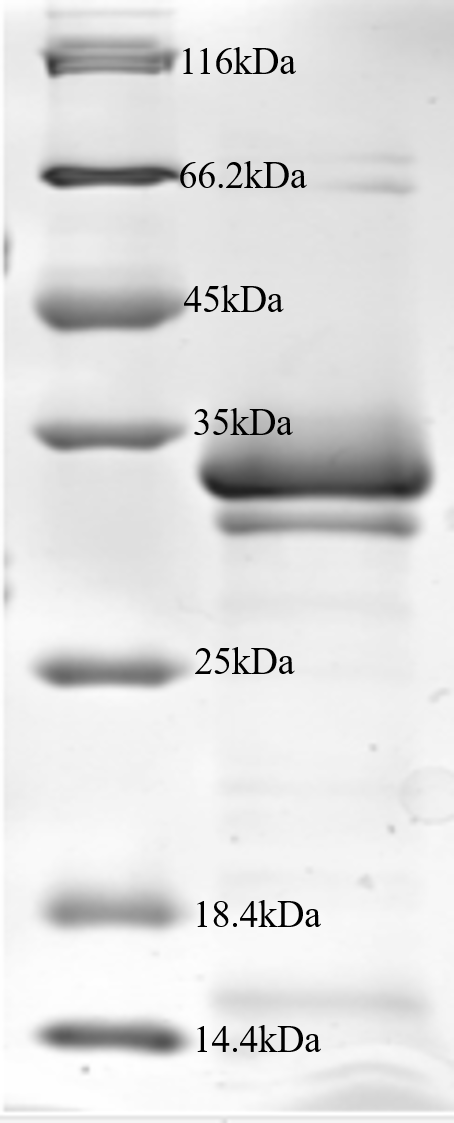

Supplement: Supplementary file 1 [file ijms-24-02207-s001.zip › supplementary Figure S1. SDS-PAGE analysis of the induced rMaOccludin protein..tif]

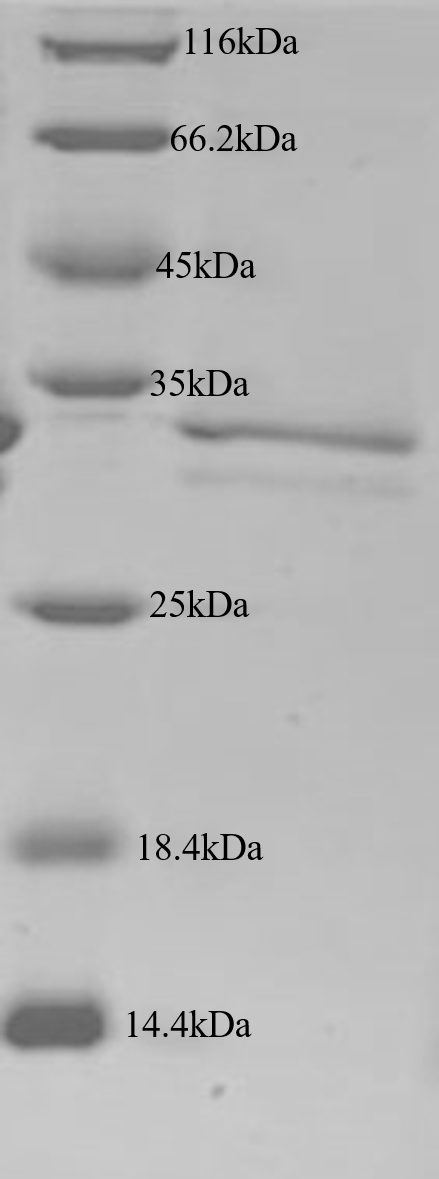

Supplement: Supplementary file 1 [file ijms-24-02207-s001.zip › supplementary Figure S2. Specificity analysis of the prepared MaCD68 antibody by Western blotting..tif]
